# EFFECT OF LONG-TERM CALORIC RESTRICTION ON THE PACE OF BIOLOGICAL AGING IN HEALTHY ADULTS FROM THE CALERIE TRIAL

**DOI:** 10.1093/geroni/igad104.0677

**Published:** 2023-12-21

**Authors:** Daniel Belsky

**Affiliations:** Columbia University, New York City, New York, United States

## Abstract

The geroscience hypothesis proposes that therapy to slow or reverse molecular changes that occur with aging can delay or prevent multiple chronic diseases and extend healthy lifespan. Caloric restriction (CR), defined as lessening caloric intake without depriving essential nutrients, can change molecular processes associated with aging, including DNA methylation (DNAm), and increases healthy lifespan in multiple species. Here we report results of a post-hoc analysis of the influence of CR on DNAm measures of aging in blood samples from the Comprehensive Assessment of Long-term Effects of Reducing Intake of Energy (CALERIE) trial, a randomized controlled trial in which n=220 non-obese adults were randomized to 25% CR or ad libitum control diet for 2years. We found that CALERIE intervention slowed the pace of aging, as measured by the DunedinPACE DNAm clock, but did not lead to significant changes in PhenoAge- or GrimAge-clock-measured biological age. Treatment effects were small; changes in DunedinPACE suggest a ~3% slowing of the pace of aging. Nevertheless, this small effect may be substantive; in an independent study of older adults, 3% slower DunedinPACE is associated with a 15% lower risk of death. The finding that CR modified DunedinPACE in a randomized controlled trial supports the geroscience hypothesis, building on evidence from small and uncontrolled studies and contrasting with reports that biological aging may not be modifiable. Ultimately, a conclusive test of the geroscience hypothesis will require trials with long-term follow-up to establish effects of intervention on primary healthy-aging endpoints, including incidence of chronic disease and mortality.

